# Combined and hybrid marker models for radiostereometry assessment of polyethylene liner motion in dual mobility hip prosthesis: a proof-of-concept study

**DOI:** 10.1186/s41747-021-00253-x

**Published:** 2021-12-15

**Authors:** Peter Bo Jørgensen, Bart L. Kaptein, Kjeld Søballe, Stig S. Jakobsen, Maiken Stilling

**Affiliations:** 1 Department of Orthopaedics, Aarhus University Hospital, Palle Juul-Jensens Boulevard 99, J118-119, 8200 Aarhus, Denmark; 2 Department of Orthopaedics, Leiden University Medical Center, 2333 Leiden, ZA The Netherlands

**Keywords:** Hip prosthesis, Phantoms (imaging), Polyethylene, Radiography, Radiostereometric analysis

## Abstract

**Background:**

Investigation of polyethylene liner movement in total hip arthroplasty requires bead-marking for radiographic visibility of the liner. However, occlusion of markers poses a challenge for marker registration in radiographs.

**Methods:**

The polyethylene of a dual mobility acetabular system was marked with twelve 1-mm tantalum markers (four groups of three markers) using a custom-made drill guide. Liner motion in a phantom and a patient was investigated with dynamic radiostereometry analysis (dRSA) at 1-year follow-up and static radiostereometry analysis (sRSA) postoperatively and at 1- and 2-year follow-up. A combined marker configuration (CMC) model was calculated from the registered positions of the liner markers and the femoral head in several images. Furthermore, the CMC model and the theoretic marker positions from computer-assisted models of the drill guide were combined in a hybrid model.

**Results:**

The CMC model included eleven markers in the phantom and nine markers in the patient, which was sufficient for dRSA. Liner movement in the phantom followed liner contact with the femoral neck, while liner movement in the patient was independent. The hybrid model was necessary to determine liner orientation in sRSA recordings, which clearly changed from postoperative to 1- and 2-year follow-up even though the patient was positioned similarly.

**Conclusion:**

Polyethylene liner motion in dual mobility hip prosthesis can be assessed with CMC models in dRSA recordings. In sRSA, the liner position between follow-ups is unpredictable and analysis requires inclusion of all markers in the model, accomplished with a hybrid marker model.

**Trial registration:**

ClinicalTrials.gov [NCT02301182], 25 October 2015.

**Supplementary Information:**

The online version contains supplementary material available at 10.1186/s41747-021-00253-x.

## Key points

- The occluded marker problem can be solved using a combined marker configuration model.
- Combination of marker data from multiple radiostereometry recordings improves analysis.
- Combination of measured and theoretic marker data further improves analysis.

## Background

Recurrent dislocation is one of the most common reasons for revision of total hip replacement [1]. The dual mobility acetabular system is designed to reduce dislocation rate by providing increased jump distance, increased range of motion and reduced risk of impingement [2]. It has a mobile polyethylene (PE) liner that articulates with respect to both the outer metal shell and the femoral head. The movements of the liner have been investigated experimentally and in a retrieval study but no clinical assessment of dual mobility liner kinematics in patients have been performed. This is because radiographic imaging methods are challenged by PE liner radiolucency, liner symmetry and liner occlusion by metal components, bone and soft tissue [3, 4].

Dynamic radiostereometric analysis (RSA) is an accurate stereoradiography method that records several radiograph pairs (frames) per second. The method allows for kinematic analysis of joints by use of bone models, implant models and marker models of tantalum markers inserted in the bones [5–9]. Formerly, dynamic RSA has been used to investigate native hip joint and total hip arthroplasty kinematics and pathomechanics, while static RSA has been used to measure polyethylene liner wear in single mobility total hip arthroplasty by insertion of tantalum markers in the PE [10, 11].

**Fig. 1:**
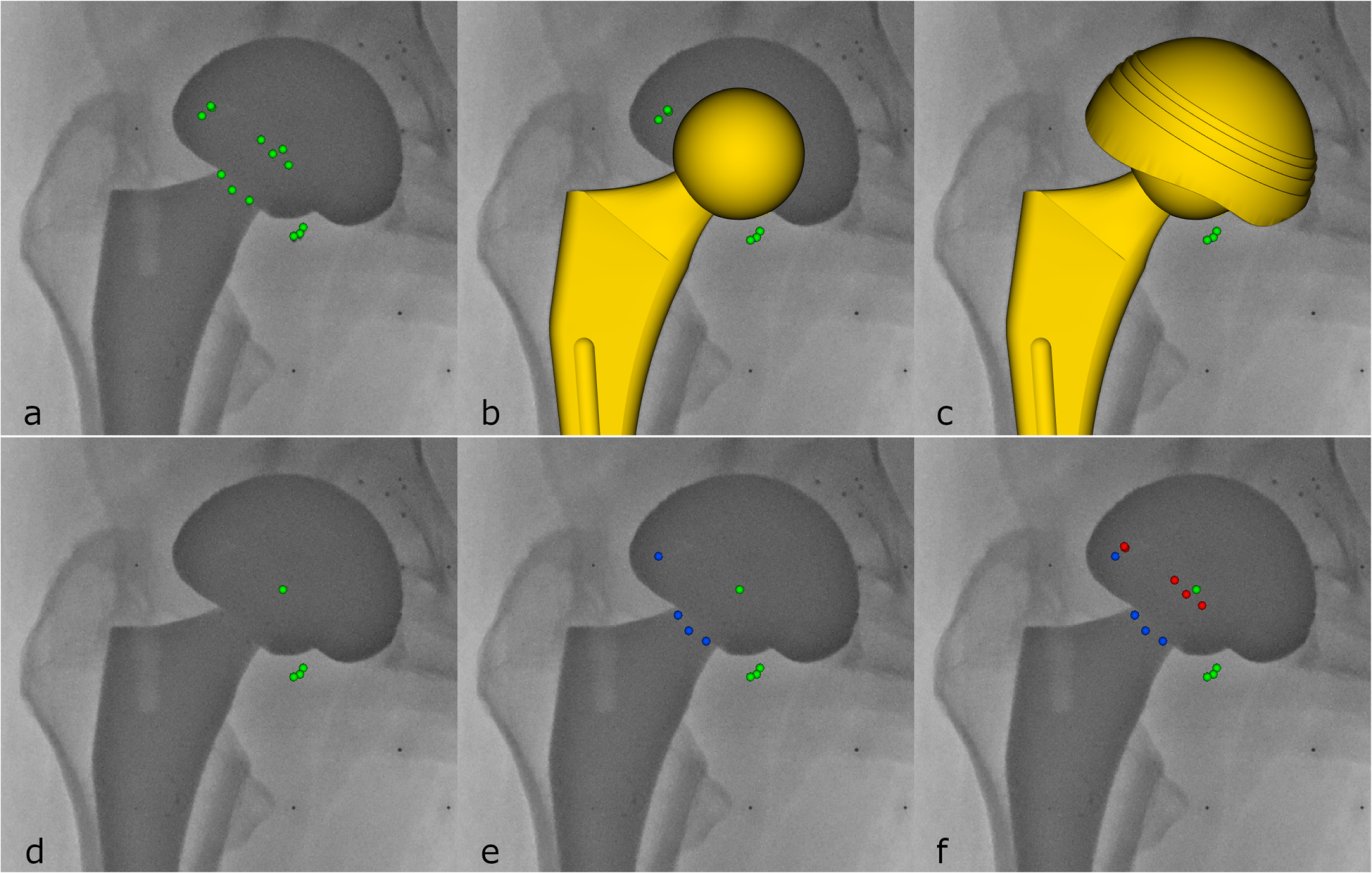
The occlusion of liner markers by the shell and the head/neck/stem (only the left radiostereometric analysis image frame is shown). Despite a high number of markers in the liner (*n* = 12) (**a**), they tend to be occluded by the head/neck (**b**) and cup (**c**) in RSA recordings. Marker information can be used as a simple marker model (green markers) (**d**), a combined marker configuration model (**e**) that merges marker information from several recordings (blue markers) or a hybrid model (**f**) that adds the theoretic marker positions (red markers) from the computer-assisted drawings of the drill guide used to insert the tantalum markers

When tantalum markers are inserted in a dual mobility PE liner, the three-dimensional position for each marker can be calculated with RSA when the marker is visible in the radiographic image pair. This may allow for kinematic analysis of the PE liner using dynamic RSA. However, when one of the two projections of a marker is not visible in the radiographic image pair, the three-dimensional position of that marker cannot be calculated (Fig. 1). A marker configuration model does not need all markers projected on both radiographs [8]. By combining information on marker positions from multiple RSA frames into a combined marker configuration (CMC) model, we aimed to build the most complete marker configuration model for the individual patient to solve the problem of marker occlusion and marker/liner position change during motion. By subsequently expanding the CMC model with theoretical marker positions, we aimed to create a hybrid model that included all liner markers and had the highest probability of precise assessment of PE liner kinematics.

The purpose of the study was to generate and test a CMC model and a hybrid model for the assessment of PE liner motion with dynamic and static RSA in a phantom and a patient.

## Methods

The study used a phantom set-up for method development and evaluated the CMC and hybrid models in a female patient (65 years old, with body mass index 32.6). The patient was recruited from a randomised clinical trial (Clinical Trial NCT02301182) and had consented orally and in writing to study participation. The Helsinki II declaration was followed [12].

### Implants and surgery

The Anatomic Dual Mobility Restoration acetabular system (Stryker, Warsaw, Mazovia, Poland) with a mobile liner made of X3 highly cross-linked PE (Stryker, Warsaw, Mazovia, Poland) and a ceramic size 28-mm femoral head was used in both the phantom and the patient. The hip stems were a Bi-Metric size 7 (Biomet, Warsaw, IN, USA) in the phantom and Accolade II (Stryker, Warsaw, Mazovia, Poland) size 4 in the patient. Cup/liner 56-mm size was used in the phantom and 50-mm size was used in the patient. An experienced hip surgeon inserted the components into the Sawbone hip (No 1301-165-1, Sawbones, WA, USA) and also the patient by use of a posterolateral approach.

### Insertion of markers in the polyethylene

**Fig. 2:**
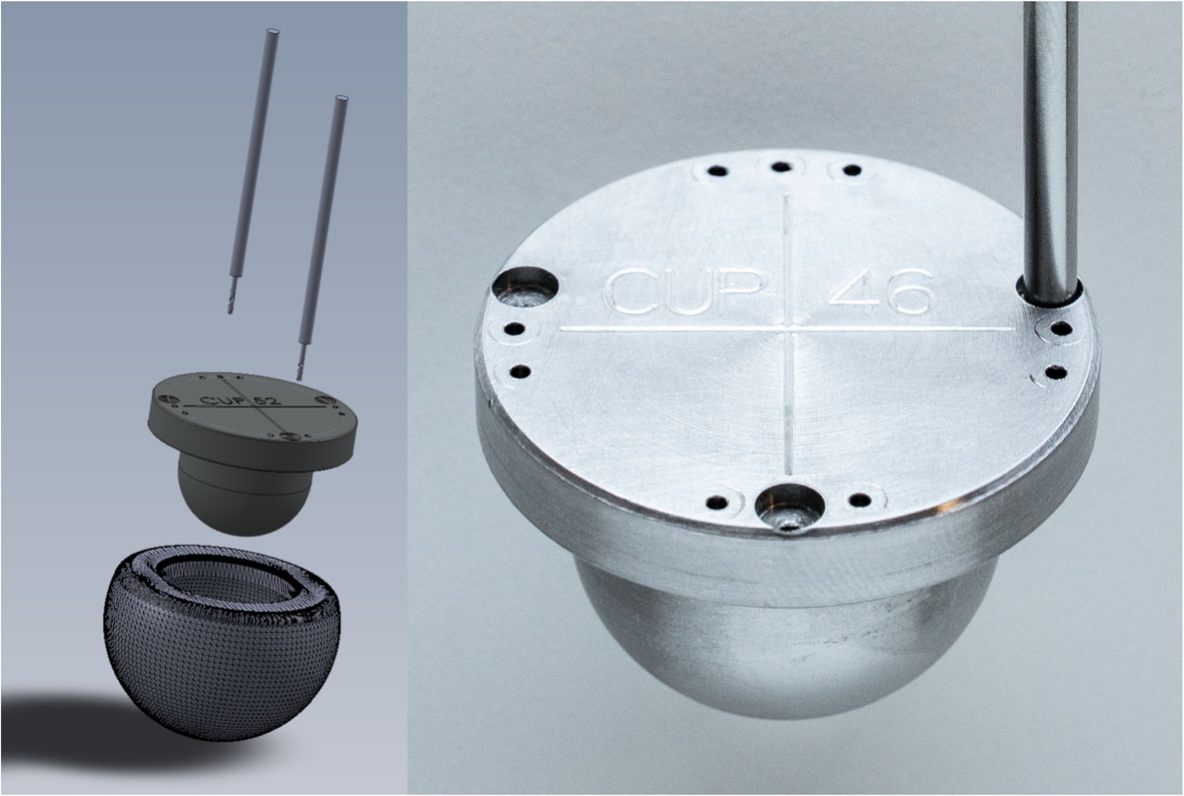
A customised tool with a drill guide for inserting markers in the individual liner sizes of the system was developed (left) and machined in stainless steel (right). Three markers with increased depth ensured distinctive marker groups

Twelve 1-mm tantalum markers (X-medics, Frederiksberg, Denmark) were placed centralised in the PE wall of the mobile liners in four groups of three markers by use of a custom-made drill guide, specific for each liner size (Figs. 1 and 2). In three of the four marker groups, one specific marker was placed 1.5 mm deeper than the other two markers, which provided a recognisable and unique pattern for each marker group.

### Radiostereometric recordings

**Fig. 3:**
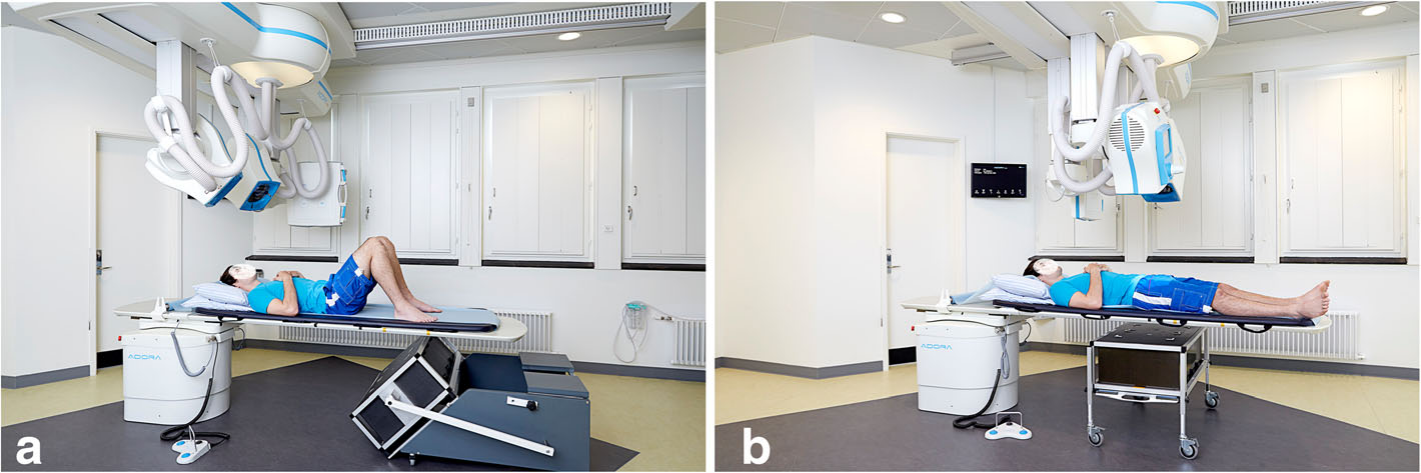
Radiostereometric analysis set-up with a direct anteroposterior angle for static recordings (**a**) and a 45-degree recording angle (**b**) for dynamic recordings in order to obtain an optimal view of the polyethylene liner

The RSA recordings were obtained using the AdoraRSA Suite (Nordic X-ray Technique, Hasselager, Aarhus, Denmark) consisting of two ceiling fixed x-ray tubes angled 40° on each other. For static RSA, two static digital detectors (CXDI-70C, Canon, Tokyo, Japan) were mounted below a calibration box (cb24, Medis Specials, Leiden, The Netherlands) for direct anterior/posterior recording (Fig. 3a).

**Fig. 4:**
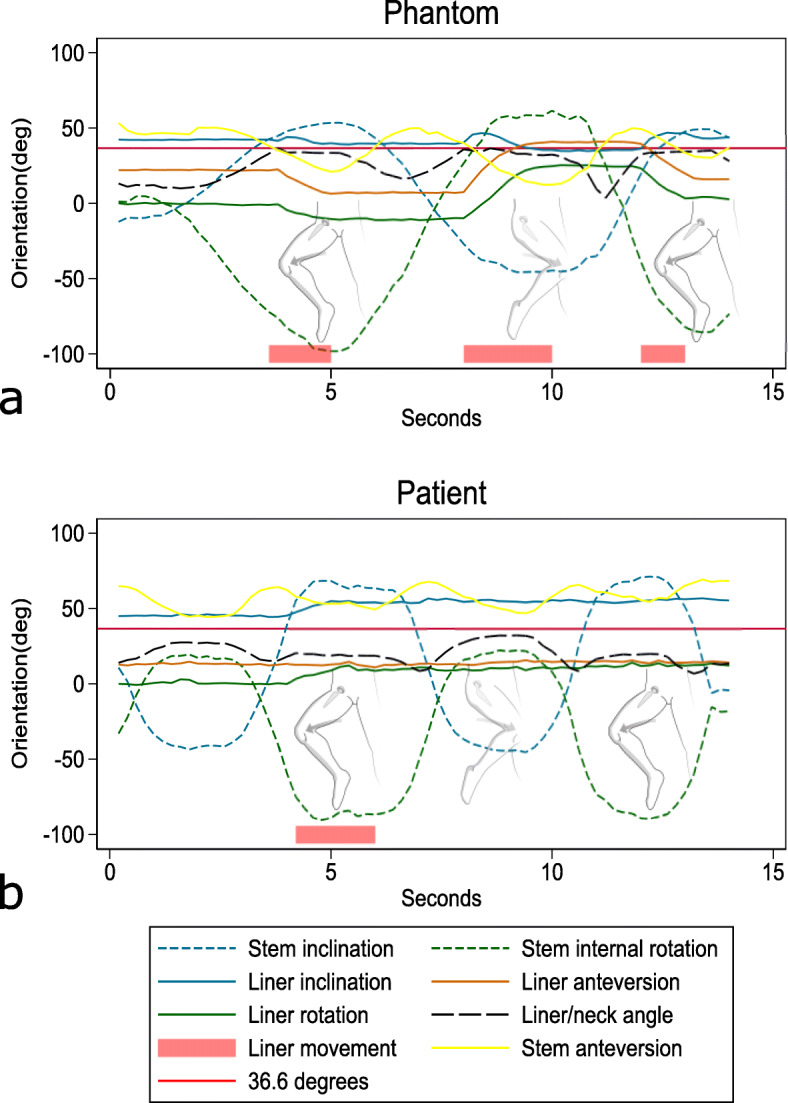
Liner rotation and stem rotation of the phantom (**a**) and patient (**b**). Dashed lines show stem movements as indicated by the pictogram. In the phantom, liner movement (solid lines) occurs in the end range of modified FABER motion (at 4 and 12 s) and of modified FADIF motion (at 8 s) when the liner/neck angle (black) approaches 36.6° (solid red). In the patient, liner movement occurs in the end range of modified FABER motion (at 4 s) without the liner/neck angle approaching 36.6°

For dynamic RSA, two dynamic digital detectors (CXDI-50RF, Canon, Tokyo, Japan) were mounted below a calibration box (cb14, Medis Specials, Leiden, The Netherlands) and recorded five images per second. From the projection of the calibration box markers, the foci position can be calculated, which enables projection of the hip implant and markers and comparison of implant component positions. The set-up allowed for a 45-degree angle on the hip joint in the cranial/caudal and anterior/posterior x-ray direction for optimal view (less marker occlusion) of the PE liner in the dual mobility cup (Fig. 3b). Soft tissue equivalence in terms of a 10-cm polymethyl methacrylate plate was placed in the recording area of the hip phantom during dynamic RSA recordings [13]. The hip was flexed to 45° and kept there while being moved in abduction/external rotation and adduction/internal rotation. This modified FADIR/FABER movement was performed passively to end-range position (Fig. 4). The dynamic RSA recordings were captured using 140 kVp and 8 mAs for the phantom and 130 kV and 8 mAs for the patient.

### Combined marker configuration model

Marker configuration model-based RSA [8] requires a marker configuration model that describes the positions of the markers in the rigid body relative to each other. By fitting this model to its projections in the RSA radiographs, the position and orientation of the model are calculated, similar to model-based RSA [8, 14]. Such a model is created from one RSA frame, in which all markers are visible in both projections, using conventional RSA [8]. The method handles the occluded marker problem, but requires that all the model markers are projected on both RSA images in one RSA frame [8]. The combined marker configuration model (CMC model) builds on the same principles, but combines the marker positions and the position of the femoral head from more than one RSA frame in the model.

Combining two or more RSA frames requires at least three overlapping markers in the image pairs. By using the femoral head as one common marker in the marker model, the minimum number of overlapping markers needed for combining RSA frames is reduced to two.

For the phantom, only dynamic RSA frames were used to generate the CMC model. For the patient, dynamic hip RSA frames were combined with standard supine static hip RSA recordings to generate a CMC model with a sufficient number of representative markers.

For creating the CMC model, the detected markers of all frames were aligned using the migration function of the mbRSA software (version 4.2, RSAcore, Leiden, The Netherlands). For both the phantom and the patient, the three-dimensional marker coordinates were exported and the mean marker positions were calculated using a custom-made program in MatLab (version 2019b, The MathWorks Inc, Natick, MA). To evaluate the dispersion of markers contributing to the CMC model, we used the standard deviation of the aligned marker positions [15].

### Hybrid marker model

A hybrid model was created by combining the marker registrations in the mean CMC model and the theoretic marker positions known from the computer-assisted drawings of the drill guide. The theoretic marker positions were aligned with the mean CMC model to add more information to the model and to be able to detect the specific four marker groups in the liner for precise registration of liner rotation. The hybrid model was used for detecting liner movement in static RSA follow-ups over time, where the liner rotation could be very different from one RSA recording to the next.

### Coordinate systems

**Fig. 5:**
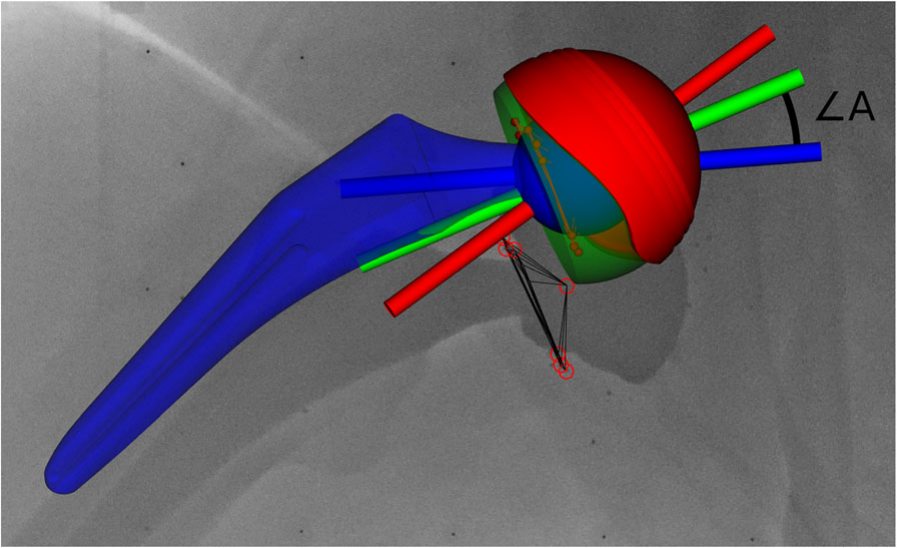
Example of anatomic dual mobility cup with a combined marker configuration model. The *y*-axis is shown for the femoral neck (blue), the cup (red) and the liner (green). ∠*A* indicates the liner/neck angle. The red circles indicate the detected marker projections in the image, as well as the centre of the femoral head

To define the local coordinate system of the CMC model, a base plane was fitted through the markers in the liner and the local coordinate system was redefined with the femoral head as origin and the *y*-axis perpendicular to the base plane of the liner. The coordinate system for the theoretic markers was created in a similar fashion, but the base plane excluded the three markers that were deeper in the liner wall. The hybrid model inherited the theoretic coordinate system. For the outer metal cup, a similar local coordinate system was defined with the origin in the centre of rotation of the cup and the *y*-axis (acetabular axis) perpendicular to the base plane of the cup. Lastly, the femoral neck coordinate system was defined with the femoral head as the centre and the *y*-axis aligned with the neck. This aligned the origins of the CMC model, the femoral head and the cup coordinate systems. In the “neutral” orientation, also the main *y*-axis of all objects was aligned. Therefore, all movements in the cup-liner-neck complex could be expressed by the angle between, *e.g.,* the cup *y*-axis and the liner *y*-axis (Fig. 5).

### RSA and data postprocessing

**Fig. 6:**
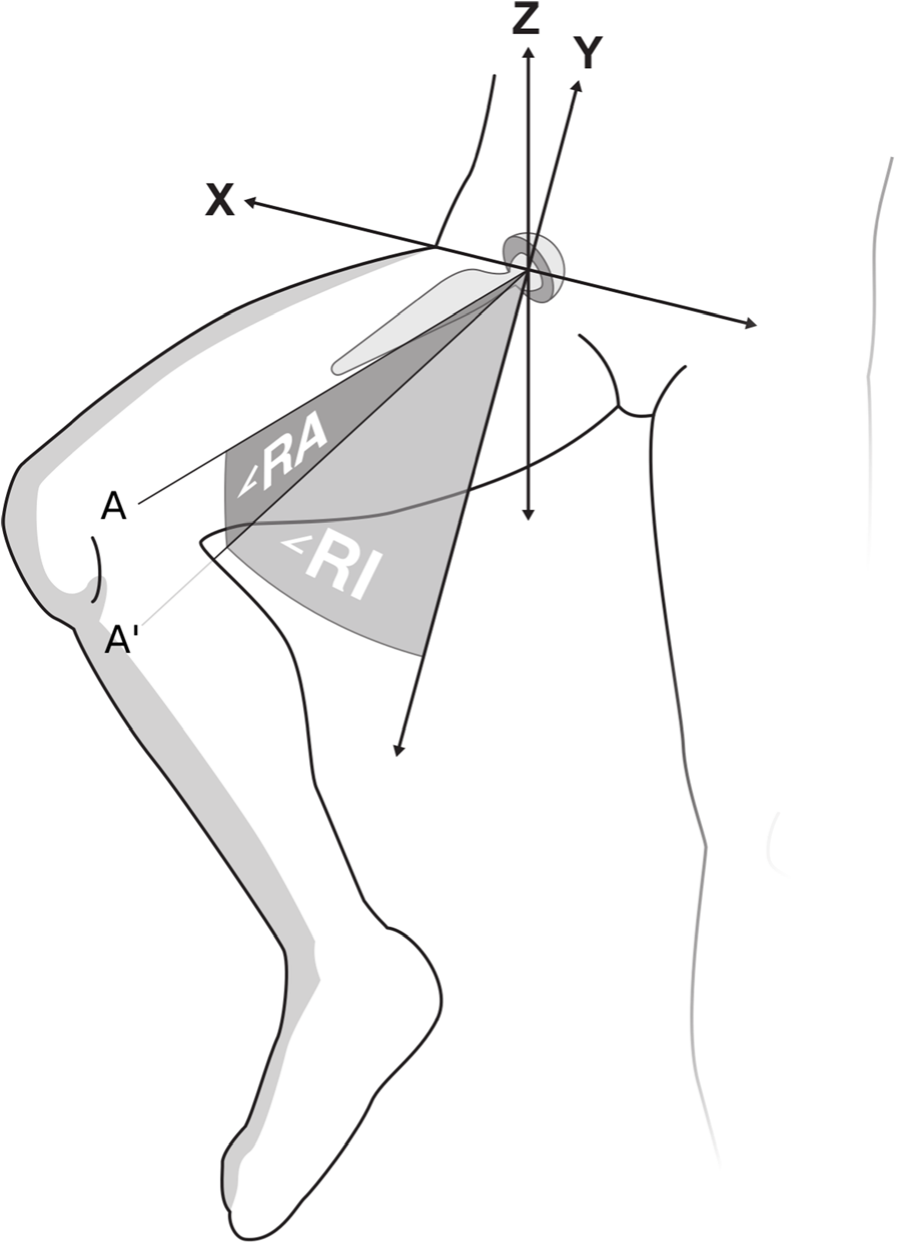
Radiographic anteversion and inclination of the cup. *A* Acetabular axis. *A’* Projected acetabular axis. ∠RI indicates the radiographic inclination, defined as the angle between the longitudinal axis (Y) and the acetabular axis (norm of cup) projected perpendicular on the coronal plane (A’). ∠RA indicates the radiographic anteversion, defined as the angle between the acetabular axis (A) and the coronal plane. X, Y, and Z represent the coordinate system of the radiostereometric analysis recording

The CMC model was then fitted to the dynamic RSA recording, frame by frame, using mbRSA (version 4.2, RSA*core*, Leiden, The Netherlands). The cup, liner- and femoral neck orientations were imported from mbRSA to a custom-made program in Python 3 [16]. To remove the patient movements during RSA recording, the raw-data orientation was standardised using the cup orientation relative to the calibration box from the first frame. This resulted in a constant cup orientation during the whole movement. The liner rotation was set to zero for the first frame of the recording. Orientation was described as inclination, anteversion and rotation in a radiographic coordinate system as described by Murray [17]. The radiographic inclination was defined as the angle between the longitudinal axis and the acetabular axis when projected on the coronal plane. Likewise, the radiographic anteversion was defined as the angle between the acetabular axis and the coronal plane [17] (Fig. 6). Stem angles were likewise calculated as standardised radiographic inclination and anteversion. Furthermore, the angle between the neck and liner normal (*y*-axis) was calculated (Fig. 5). This angle served as an indicator of contact between the neck and the liner. With contact between neck and liner, the liner should rotate relative to the cup. Movements were graphically displayed using Stata/IC (version: 16.0, StataCorp, College Station, TX, USA).

## Results

The phantom RSA frames had four common conventional markers and the patient frames had two common conventional markers. All frames had additional markers that could not be used in the standard analysis as they did not represent the same markers between frames. The CMC model for the phantom was derived from five dynamic RSA frames and comprised of eleven markers (including the femoral head). The CMC model for the patient was derived from three dynamic RSA frames and one static RSA frame and comprised of nine markers. The maximum standard deviation of marker positions occurred in the out-of-plane *z*-direction for both the phantom and the patient (Tables 1 and 2).

**Table 1:** Markers represented in the combined marker configuration model of the phantom and the patient as specified by marker ID

	Phantom				Patient			
Marker ID	*x*-axis (*SD*)	*y*-axis (*SD*)	*z*-axis (*SD*)	Number of markers*	*x*-axis (*SD*)	*y*-axis (*SD*)	*z*-axis (*SD*)	Number of markers*
1	0.04	0.06	0.09	5				
2	0.05	0.08	0.19	5				
3	0.07	0.11	0.06	5				
4					0.25	0.19	0.11	4
5	–	–	–	1	0.13	0.14	0.28	3
6	0.12	0.11	0.01	2	0.10	0.18	0.26	3
7	0.09	0.07	0.10	3	0.03	0.35	0.16	2
8	0.12	0.02	0.29	2	0.14	0.15	0.28	2
9	–	–	–	1	0.08	0.04	0.21	2
10								
11	0.03	0.09	0.36	2	0.07	0.56	0.09	2
12	–	–	–	1	–	–	–	1
Femoral head	0.14	0.13	0.14	5	0.28	0.21	0.21	4

*Number of markers available to calculate the marker position in the combined marker configuration model. *Marker ID* ID number of the marker in the model, *SD* Standard deviation of marker position per (calibration box) axis of the radiostereometric analysis frame to which the markers were aligned

**Table 2:** Representation of markers in individual recordings

		Phantom					Patient			
Recording		1	2	3	4	5	1	2	3	4
Conventional markers	All recordings	4	4	4	4	4	2	2	2	2
	Additional markers	2	3	4	3	1	4	2	5	4
Combined marker configuration model	Number of markers in the model	11	11	11	11	11	9	9	9	9
	Left	10	8	10	11	7	6	7	7	6
	Right	9	8	9	3	9	6	6	7	7

The phantom frames have four common conventional markers and the patient frames have two common conventional markers. All frames have additional markers that cannot be used in the standard analysis as they do not represent the same markers between frames. With the combined marker configuration model, eleven markers were utilised in the phantom and nine in the patient; all projections of these markers were available for analysis. *Conventional markers* Markers clearly visible in both images of the frame, *Additional markers* Markers that are visible in both images of the frame but cannot be included in a standard analysis as they do not represent the same marker between frames, *Combined marker configuration model* Markers and marker projections used for fitting the combined marker configuration model

### Phantom

The phantom-liner was positioned neutral to the cup opening, and liner motion started when the liner/neck angle approached 36.6° during the modified FABER motion, which is the angle of contact between the liner and the neck (Fig. 4, 4 s). Reversely, at the start of the modified FADIR motion, the liner/neck angle fell below 36.6° and the liner stopped moving until about halfway through the modified FADIR motion, when the liner/neck angle again reached 36.6°, and the neck contacted the liner and initiated movement. Liner movement was primarily caused by contact with and pushing from the femoral neck at 36.6 liner/neck angle; however, some spontaneous motion also occurred at lower liner/neck angles between 32.9 and 36.6° (Fig. 4). The range of stem movement during recording was 99° inclination, 41° anteversion and 160° rotation. The range of liner movement was 12.2° inclination, 35° anteversion and 37° rotation. The range of liner/neck angle was 33°.

### Patient

With dynamic assessment, the liner started in a position of 45° inclination and 12.8° anteversion and started to move when the stem reached the maximum modified FABER motion for the first time (Fig. 4b, 4 s). Although a slight liner movement happened at 0° stem inclination (Fig. 4b, 7 s), the liner remained stable during the second modified FABER motion. Liner movement was not caused by contact with the femoral neck as the liner/neck angle was higher in the FADIR motion (without liner movement) compared to the FABER motion (Fig. 4b). The ranges of hip stem movement during dynamic RSA recording were 117° inclination, 25° anteversion and 113° rotation. The ranges of liner movements were 12° inclination, 5° anteversion and -15° rotation (Fig. 4b). The range of liner/neck angle was 24°.

Static RSA evaluations at postop and at 1- and 2-year follow-up were completed with the hybrid model (all markers), which enabled registration of the liner orientation despite substantial and unpredictable liner rotation between follow-ups. Liner inclination was relatively stable from baseline to 1- and 2-year follow-up. Anteversion decreased from 12 to 9° and 0° and rotation was measured to -109°, -133° and -141° (Table 3).

**Table 3:** Static liner orientation of the patient at postoperative baseline, 1- and 2-year follow-up

	Baseline	1-year	2-year follow-up
Inclination	48°	54°	48°
Anteversion	12°	9°	0°
Rotation	-109°	-133°	-141°

Liner orientation measured in the coordinate system of the calibration box at baseline and adjusted for the cup orientation in subsequent follow-ups

## Discussion

This study is the first to quantify *in vivo* PE liner motion of a dual mobility PE in total hip arthroplasty. The study demonstrates the feasibility of a CMC model, which combines registration of the femoral head with markers inserted in the PE liner, and a hybrid marker model, which combines the CMC model with the theoretical marker position in the PE.

### Utilising the femoral head as a marker

In this study, the femoral head was utilised as a marker in the CMC model. This proved to be a great advantage since the femoral head is very likely to be detectable in RSA recordings. The theoretic disadvantage is that the femoral head and the liner are not the same rigid body. In the ADM cup, the femoral head and the liner are joined with a press fit, and very unlikely to sub-lux. Still, microtranslations are possible, and over time also PE wear may compromise the use of the femoral head in a CMC model. Nevertheless, using the femoral head in the model enabled analysis of RSA frames that would have been impossible to analyse with standard methods.

### The strengths and weaknesses of the CMC model

The CMC model builds on mean marker positions from multiple RSA frames, which in theory reduces the random error in the position of the markers. However, the use of mean marker positions in the model makes it inherently sensitive to the inclusion of markers with few data points and a large variation where one outlier can have a great impact on the mean value. The standard deviation of the mean marker position is a summary measure of this variation.

The use of a CMC model enables fitting of the model with a minimum of markers in the RSA recording. This is a great advantage when image quality is compromised and marker visibility, marker detection and marker model creation by standard algorithms is not possible [8]. In fact, a marker configuration model requires as little as four marker projections of the model (3 projections in one image and 1 projection in the other image) in the RSA recording to enable a clinical meaningful calculation [8]. Furthermore, the robustness makes the marker configuration model useful for the assessment of PE wear or liner motion in knee as well as hip arthroplasty [18, 19]. The use of a CMC model solves the occluded marker problem in dynamic RSA recordings where different markers are visible in a series of RSA frames.

### The strengths and weaknesses of the hybrid model

Adding theoretic marker positions to the CMC model introduces a new source of error as well as valuable information. Lam-Tin Cheung et al. [20] showed good results with theoretic markers when using a drill guide for marker placement. The disadvantage is that the validity of theoretic markers relies heavily on knowledge of the initial marker position and subsequent marker migration. The great advantage of theoretic markers is that information from just a single marker projection in any of the images of the RSA recordings can add to the analysis. The model should be used when the completeness of the model outweighs the risk of misplaced markers.

### Quality parameters in marker configuration models

Condition number and mean error of rigid body fitting are quality parameters that should be used to verify standard RSA results. The condition number is a mathematical expression of how close the markers in the model are located on a line [21]. The upper acceptance limit for condition number is 150 for hip and knee RSA [22]. When using marker configuration models, the condition number indicates the marker distribution in the marker configuration model but does not describe the marker distribution in the individual frame.

The mean error of rigid body fitting indicates variation in relative position of markers between RSA frames. In a standard marker-based RSA analysis with the mbRSA software, markers that cause a variation in average relative position larger than 0.35 mm are discarded [22, 23]. This is not the case with marker configuration models: analysis of RSA recordings in mbRSA software will obtain the best possible fit of a markers model disregarding eventual changes in marker positions (*e.g.,* migrating/loose makers). Therefore, careful manual/visual quality assurance should be performed when using marker configuration models.

### Alternatives to CMC and hybrid models

Alternative approaches for liner tracking have been described. Zaribaf et al. [24] investigated the possibility of adding a radiopaque medium to PE, to make it visible on a standard radiograph. Although this method showed promising results as the material became radiopaque and maintained good strength, acetabular liners are symmetric and rotations are therefore difficult to visualise and quantify with RSA. Also, the technique was not tested in a clinical setting. For knee implants, the PE liner motion has been tracked indirectly assuming that it fills the space between the femoral and tibial components when these are of a congruent design [25].

### Feasibility and biomechanical outcome

In this study, the analysis using the CMC model was sufficient to analyse dynamic RSA recordings with only little and predictable liner motion between recorded image frames. Combining data on neck, stem and liner motion revealed the interaction of these components to initiate liner motion. The motion pattern of all components could be graphically outlined and document liner motion *in vivo*. In the phantom, the measured liner motion followed an expected pattern of movement when the neck contacted the liner at the 36.5° liner/neck angle [26]. In the patient, the liner/neck angle was considerably lower during liner movement, which shows that movement in the liner can also occur without liner/neck contact. Also, the total liner motion in the patient was less than in the phantom. Expectedly, the explanation for liner movement without neck contact is soft tissue contact. Thereby, soft tissue contact with the PE liner *in vivo* can also be a restraint for the liner to position safely. Likely, this makes the liner motion patterns and polyethylene wear areas very heterogeneous between patients. This is the first time that liner motion of a dual mobility PE liner has been quantified *in vivo* and more clinical data is necessary to further investigate liner motions *in vivo*.

For analysis of liner motion in static images *in vivo*, the liner position was unpredictable between follow-ups. In this case, the hybrid model ensured identification of the four unique marker groups in the liner and enabled analysis of liner position. Static analysis over time revealed large rotations and smaller changes in inclination and anteversion. Because of the large rotation of the liner, analyses were only possible due to the completeness of the hybrid model.

In conclusion, PE liner motion in dual mobility hip prosthesis can be assessed in dynamic RSA recordings with CMC models that are reconstructed from marker positions in multiple RSA recordings. The liner position between yearly follow-ups is unpredictable and analysis requires the inclusion of all markers in the PE liner model, which can be accomplished with a hybrid marker model that combines both the registered positions of visible markers and the theoretical position of occluded markers. The method was developed specifically to enable an analysis of a mobile PE liner in a dual mobility cup, but the concept can be applied in any static or dynamic RSA analysis complicated with altering marker visibility on successive frames.

## Supplementary Information

**Additional file 1.**

**Additional file 2.**

**Additional file 3.**

## Data Availability

The datasets generated and/or analysed during the current study are not publicly available due to the sensitive nature of radiographic images but are available from the corresponding author on reasonable request.

## Abbreviations

CMC: Combined marker configuration
PE: Polyethylene
RSA: Radiostereometric analysis

## References

[1] DHR (2019). National report 2019, The Danish Hip Arthroplasty Register, Regionernes Kliniske Kvalitetsudviklingsprogram.

[2] Blakeney WG, Epinette JA, Vendittoli PA (2019). Dual mobility total hip arthroplasty: should everyone get one?. EFORT Open Rev.

[3] D'Apuzzo MR, Koch CN, Esposito CI (2016). Assessment of damage on a dual mobility acetabular system. J Arthroplasty.

[4] Grazioli A, Ek ET, Rudiger HA (2012). Biomechanical concept and clinical outcome of dual mobility cups. Int Orthop.

[5] Hansen L, De Raedt S, Jorgensen PB (2018). Marker free model-based radiostereometric analysis for evaluation of hip joint kinematics: a validation study. Bone Joint Res.

[6] Digas G, Johansson PE, Karrholm J (2013). Inducible displacements of the cup and the femoral head during active range of motion: dynamic RSA studies of cemented total hip replacements. J Orthop Res.

[7] Zugner R, Tranberg R, Lisovskaja V, Shareghi B, Karrholm J (2017). Validation of gait analysis with dynamic radiostereometric analysis (RSA) in patients operated with total hip arthroplasty. J Orthop Res.

[8] Kaptein BL, Valstar ER, Stoel BC, Rozing PM, Reiber JH (2005). A new type of model-based Roentgen stereophotogrammetric analysis for solving the occluded marker problem. J Biomech.

[9] Baad-Hansen T, Kold S, Kaptein BL, Soballe K (2007). High-precision measurements of cementless acetabular components using model-based RSA: an experimental study. Acta Orthop.

[10] Borlin N, Rohrl SM, Bragdon CR (2006). RSA wear measurements with or without markers in total hip arthroplasty. J Biomech.

[11] Nebergall AK, Rader K, Palm H, Malchau H, Greene ME (2015). Precision of radiostereometric analysis (RSA) of acetabular cup stability and polyethylene wear improved by adding tantalum beads to the liner. Acta Orthop.

[12] World Medical Association (2013). World Medical Association Declaration of Helsinki: ethical principles for medical research involving human subjects. JAMA.

[13] Mann KS, Kurudirek M, Sidhu GS (2012). Verification of dosimetric materials to be used as tissue-substitutes in radiological diagnosis. Appl Radiat Isot.

[14] Kaptein BL, Valstar ER, Stoel BC, Rozing PM, Reiber JHC (2003). A new model-based RSA method validated using CAD models and models from reversed engineering. J Biomech.

[15] ISO (2019). International ISO standard ISO 5725: accuracy (trueness and precision) of measurement methods and results.

[16] GaD VR, Fred L (2009). Python 3 Reference Manual.

[17] Murray DW (1993). The definition and measurement of acetabular orientation. J Bone Joint Surg Br.

[18] Trozzi C, Kaptein BL, Garling EH (2008). Precision assessment of model-based RSA for a total knee prosthesis in a biplanar set-up. Knee.

[19] Garling EH, Kaptein BL, Geleijns K, Nelissen RG, Valstar ER (2005). Marker configuration model-based roentgen fluoroscopic analysis. J Biomech.

[20] Lam-Tin-Cheung K, Yuan X, Nikolov HN (2017). Marker-based technique for visualizing radiolucent implant components in radiographic imaging. J Orthop Res.

[21] Ryd L, Yuan X, Lofgren H (2000). Methods for determining the accuracy of radiostereometric analysis (RSA). Acta Orthop Scand.

[22] Valstar ER, Gill R, Ryd L (2005). Guidelines for standardization of radiostereometry (RSA) of implants. Acta Orthop.

[23] ISO (2013). International standard ISO 16087:2013. Implants for surgery — Roentgen stereophotogrammetric analysis for the assessment of migration of orthopaedic implants.

[24] Zaribaf FP, Gill HS, Pegg EC (2020). Characterisation of the physical, chemical and mechanical properties of a radiopaque polyethylene. J Biomater Appl.

[25] Horsager K, Kaptein BL, Jorgensen PB, Jepsen CF, Stilling M (2018). Oxford medial unicompartmental knees display contact-loss during step-cycle motion and bicycle motion: a dynamic radiostereometric study. J Orthop Res.

[26] Noyer D, Caton JH (2017). Once upon a time.... Dual mobility: history. Int Orthop.

