# Supplementary figures and images for: Combined and hybrid marker models for radiostereometry assessment of polyethylene liner motion in dual mobility hip prosthesis: a proof-of-concept study
Source: Eur Radiol Exp. 2021 Dec 15;5:55. doi: 10.1186/s41747-021-00253-x (PMC8671599; doi:10.1186/s41747-021-00253-x)

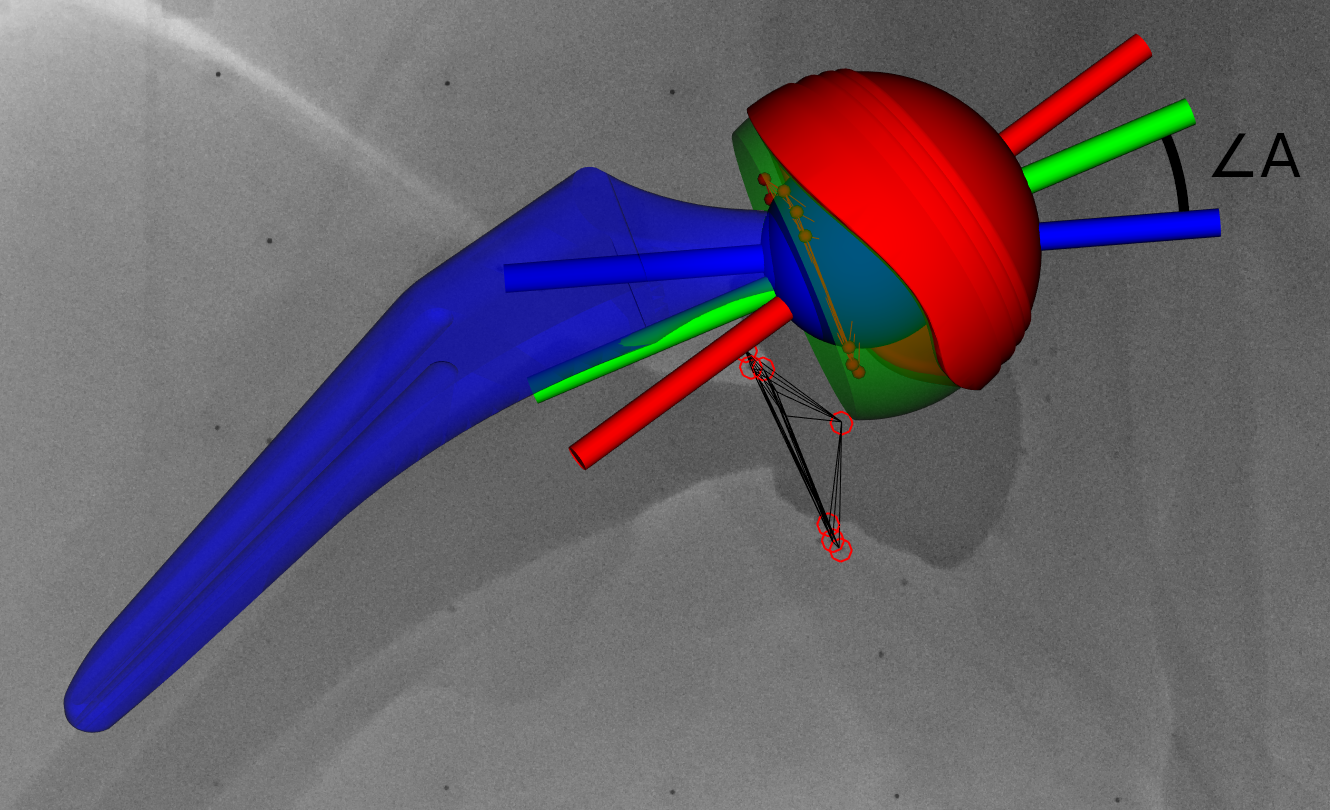

Supplement: Supplementary file 1 — Additional file 1. [file 41747_2021_253_MOESM1_ESM.tiff]
